# A 3D printed grinder adaptor for streamlined sample processing

**DOI:** 10.1016/j.ohx.2025.e00699

**Published:** 2025-09-11

**Authors:** Otto Heuschele, R. Ted Jeo, D. Jo Heuschele

**Affiliations:** USDA- ARS Plant Science Research Unit, St. Paul, MN 55108, USA

**Keywords:** Grinder adaptor, Sample transfer tool, 3D printing

## Abstract

In agricultural research, sample preparation for any type of post-harvest analysis is very time consuming. After harvesting samples, they generally need to be ground before further analysis. Grinding plant samples can be accomplished by either an abrasive type or blade type grinders. The abrasive type provides relatively uniform particle size; therefore, it is preferred to prepare material for analytical assays (1 mm). The Foss Cyclotec CT293 grinder comes with a glass container that needs to be removed and cleaned after each sample is ground. We developed a 3D printed adaptor cone that does not need to be removed after each sample and is cleaned when compressed air is vented through the machine to remove residual sample from the abrasive ring. This hardware reduces 27 % of time required for processing per sample (n = 60) and allows for direct sample grinding into different sample storage containers.

## Graphical abstract

**Figure:**
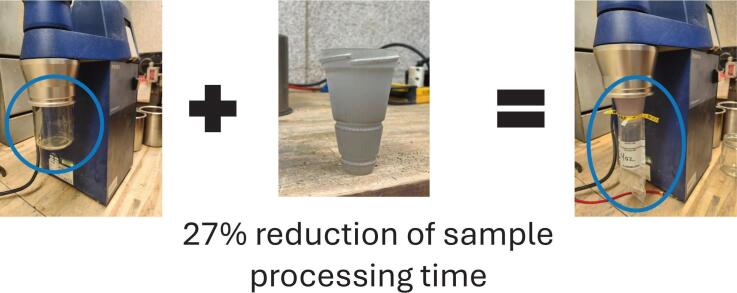

Specifications table.

**Table:** 

Hardware name	**Sample Grinder Cone Adaptor**
Subject area	• Environmental, planetary and agricultural sciences • General
Hardware type	• Biological sample handling and preparation
Closest commercial analog	No commercial analog is available.
Open-source license	CERN-OHL-P-2.0
Cost of hardware	$32.00 per unit
Source file repository	https://data.mendeley.com/datasets/npvb6k52g6/1https://doi.org/10.17632/npvb6k52g6.1

## 1 Hardware in context

The device designed was an adaptor for a grinder to be able to collect smaller samples in either generic plastic whirl or zipper type sample bags. In agricultural research, often sample sizes collected in an experiment can be very small, less than 5 g, so attention needs to be directed in ways and means to minimize the loss of these samples during processing while maintaining consistent particle sizes [1]. Additionally, where there are large numbers of samples to be ground for post-harvest analysis, hardware and protocols that reduce labor hours are essential.

**Fig. 1:**
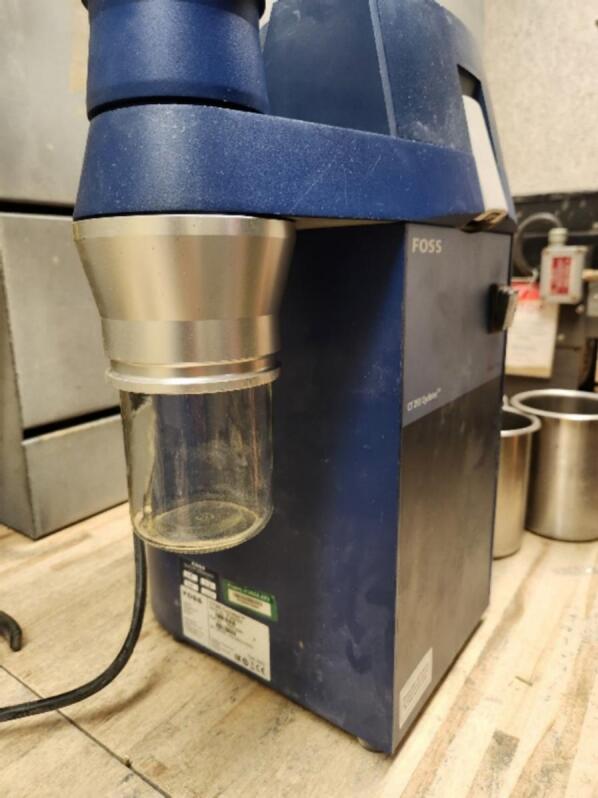
Foss CT 293 Cyclotec grinder with the glass sample jar installed. The 350 ml jar screws into machine and ground samples drop directly collected into the container. However, the sample needs to be transferred into another storage container before grinding the next sample.

The Foss CT 293 Cyclotec impeller abrasive ring grinder can grind many different types of samples to a screen specified particle size, usually 0.5 or 1 mm. The CT 293 is a research grade grinder mostly used in the agricultural research and/or product testing of food or feed samples. The grinder comes with a 350 ml glass jar used to collect the ground material that is ejected from the grinder (Fig. 1). No other container or sample transfer device by size or material is offered by the manufacturer. The standard 350 ml glass sample container is most useful when grinding 50 – 100 g of material (or a smaller number (1–10) of samples are being ground at a time. The shape and material of the container does cause ground sample material to adhere to the sides resulting in sample loss. Loss of sample can also occur when transferring the material from the jar to the storage container. To twist the jar on and off the machine to transfer material and or clean the jar takes time. This time lag is not an issue when grinding small numbers of samples, however some research labs are processing 1000′s of samples per agricultural experiment.

The research lab identified a need for an adaptor that would allow the grinding of material directly into sample storage bags of a zipper, or whirl type, to eliminate losses from the transferring sample or static cling. There was also a need to reduce the amount of work/time required to clean the standard glass jar after every sample to reduce cross contamination. An adaptor was needed that would allow a new, clean, and prelabeled sample container for each sample ground. No such device was found to be available from the manufacturer or another source.

**Fig. 2:**
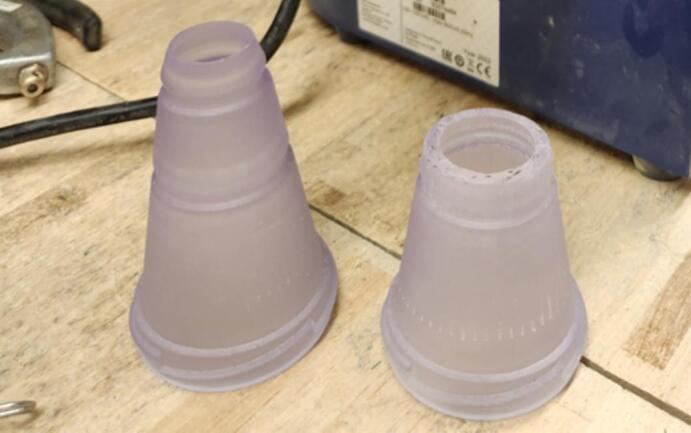
Sample adaptor designed for research grade cyclone grinders. The simple cone shape with grooves allows for an airtight seal and easy cleaning between grinding samples.

Therefore, the team designed an adaptor, in CAD (computer aided design) software and then 3D printed with a biomedical grade clear resin that is semitransparent. (Fig. 2). The adaptor replaces the original glass sample jar by attaching to the same threaded outlet. This simple adaptor has been shown to reduce the labor required to grind samples especially in research programs that need to grind large numbers of samples (i.e. > 1000) at one time.

## 2 Hardware description

**Fig. 3:**
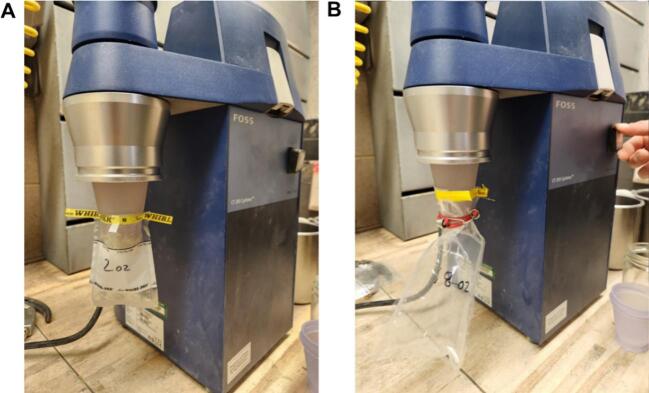
Adaptor attached to grinder. (A) The top groove has been designed for (A) 2 oz and 4 oz, while (B) the bottom groove accommodates smaller mouth sample bags or larger bags attached with a rubber cord or band.

The adaptor is a simple cone shape that can be 3D printed with a threaded top and grooves at intervals along the cone. The adaptor’s threads ensure an airtight seal and is shaped such that it can be used to attach different sized sample bags allowing smaller samples to be ground directly into very small bags without loss from container transfer (Fig. 3). The sample bags slip over the cone shaped end of the adaptor and are either held on by friction, by an elastic band or by the wire tie incorporated in whirl type bags. The device is adaptive in that it can either be 3D printed with one or two grooves on the cone to better fit different sized sample bags (Fig. 2). After each sample is ground the adaptor remains in place, unlike the manufacturer jar that is removed with each sample, and the pressurized air applied to remove any residual sample from the internal workings of the grinder, also removes residual sample from the adaptor, if any was present. The resin used in the printing of this device is biocompatible, anti-static, and inert, therefore many different materials can pass through the device without damaging the sample. This type of material will allow for the adaptor to be autoclaved if required and sample will not cling to the inner walls of the adaptor during grinding due to static build up. We have found savings in time of about 27 % per sample for our grinding protocol and a reduction of sample loss of 5 % when comparing the use of the original jar to the sample bags attached to the adaptor. Additionally, the glass jar is easily damaged or destroyed and is expensive to replace, whereas the adaptor is more resilient if detached from the machine and dropped.

Users will find:

• Little to no sample loss from transferring samples to a different container both in size and type.
• Greater throughput because containers can be pre- labeled, and samples can be ground directly into storage bags.
• Sample bags are cheaper and easier to store than the original glass jars, cutting down in storage space needed and safety requirements of fragile glass jars.

***Design files***.

All design files can be found on Mendeley at https://data.mendeley.com/datasets/npvb6k52g6/1 (https://doi.org/10.17632/npvb6k52g6.1) [2].

## 3 Design files summary

**Table:** 

**Design file name**	**File type**	**Open source license**	**Location of the file**
Two-groove Cone.obj.glb	CAD – object file	Cern-OHL-P-2.0	https://data.mendeley.com/datasets/npvb6k52g6/1
Two-groove Cone.stl	3D print file	Cern-OHL-P-2.0	https://data.mendeley.com/datasets/npvb6k52g6/1
One-groove Cone.obj.glb	CAD – object file	Cern-OHL-P-2.0	https://data.mendeley.com/datasets/npvb6k52g6/1
One-groove Cone.stl	3D print file	Cern-OHL-P-2.0	https://data.mendeley.com/datasets/npvb6k52g6/1
Quarter Turn Threads.ojb.glb	CAD – object file	Cern-OHL-P-2.0	https://data.mendeley.com/datasets/npvb6k52g6/1
Quarter Turn Threads.ojb.glb	3D print file	Cern-OHL-P-2.0	https://data.mendeley.com/datasets/npvb6k52g6/1

Files with the extension obj.glb are object files that will open on CAD or any type of CAD-like freeware. Files with the extension stl are 3D print files that can be uploaded to a 3D printer. “Two-groove Cone” files contain the design for a full- size cone including two grooves for different bag attachments. The “One-groove Cone” designs are for printing the shorter cone with only one groove. The cone with one groove can be used when the larger cone design does not allow enough room to collect sample directly below the grinder outlet. The thread design (“Quarter Turn Thread”) for the grinder has also been included with the files in case anyone would like to modify the cone design.

***Bill of materials***.

## 4 Bill of materials summary

**Table:** 

**Designator**	**Component**	**Number**	**Cost per unit −currency**	**Total cost −currency**	**Source of materials**	**Material type**
*BioMed Resin*	BioMed Clear resin	1 L	$32 per unit	11 units $350.00	Formlab formlabs.com*https://formlabs.com/store/materials/biomed-clear-resin/*	−Polymer
Isopropanol	99 % Isopropanol	1L	$ 4.00	11 units $48.00	https://www.sigmaaldrich.com	−Other (solvent)

The printing of one device does not use all the resin or isopropanol that was purchased. One liter of resin will contain enough material to print 11 devices. The isopropanol is used for removing water from the resin before curing (a soaking liquid) and can be reused for more than 11 devices. Therefore, printing a batch of 11 units reduces the unit cost to approximately $36.00 each. Any 3D printer can be used to create this device if it can print with a bio-medical type of resin. The resin was selected for the following properties: high melting temp, high impact resistance, low flexibility, can be sterilized, translucence, and biocompatibility. For the device constructed a Form3 SLA Printer (Formlab, MA, USA) was used for printing and UV sterilizer electric tool cabinet (OMWAH, WA, USA) for UV curing.

## 5 Build instructions

1. To print design, download selected open-source 3D files in STL format on Mendeley (see Design Files). The design choices are either a one or two grooved cone.
2. Obtain proper materials and equipment. For this example, a Form3 SLA Printer (Formlabs MA, USA) and BioMed Clear resin (Catalog # RS-CFG-BMCL-01) (Formlabs MA, USA) was used.
3. Input STL file into a slicer to format the document into something with “supports” in an orientation that the printer can use. The example used Form Labs 3D slicer.
4. Within the slicing software, rotate the cone model so that the narrow opening is towards the build plate of the printer for best support removal later.
5. Use the smallest layer setting possible to create a smooth finished product. This example used a 100um setting.
6. Select the smallest raft size the slicer software to reduce material waste. In this example the mini raft setting was used.
7. Save the updated file to the computer. This step will change the file format from the original format to an STL format that includes supports.
8. Upload the formatted file to the 3D Printer. Most printers require the user to select a file that has been uploaded to the printer to execute the job.
9. Print the grinder cone using the print function of the specific printer selected. Do not remove lid as the device is printing to prevent injury from the printing laser (Fig. 4).

**Fig. 4:**
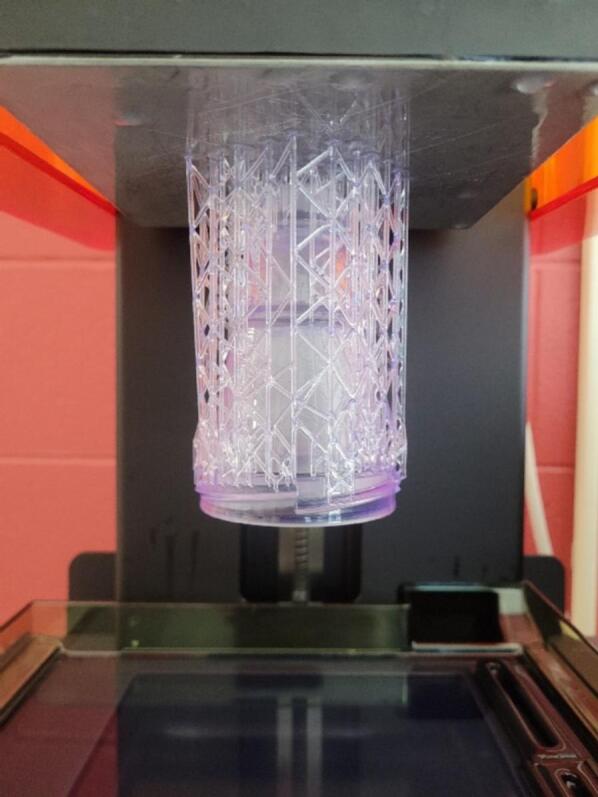
Device with printed supports on the building platform.
10. Once the print has been completed, gently remove the cone from the supports wearing gloves (Fig. 5). The resin is only biocompatible after curing.

**Fig. 5:**
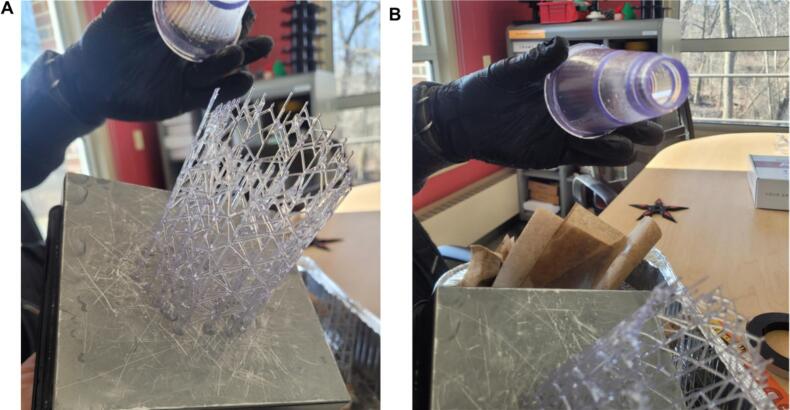
Finished printed device. A) The cone is gently removed from the supports. B) Cone is now ready to be submerged into isopropanol which removes residual resin from where the supports attached to the device.
11. Still wearing gloves, soak in 99 % isopropyl alcohol for a minimum of 15 min to assist in removal of the uncured and loose resin from the print. Isopropyl can be recycled to clean other prints and/or should be disposed of according to proper waste management practices.
12. Wipe down the outside of the print with a paper towel soaked in 99 % isopropyl alcohol until there are no drips or excess resin.
13. Put the fresh print in either a UV curing box or UV sterilizer for an hour or until fully cured and no longer tacky at room temperature. The device can now be handled without gloves.
14. Leave fresh print in direct sunlight for 8 h to off gas and further cure. This product may need up to 48 h to fully harden before it can be put in an autoclave. This product will NOT over-cure.

## 6 Operation instructions

Note that the threads on this adaptor were designed for Foss CT 293 Cyclotec grinder. Basic safety procedures should be followed whenever using any type of grinder: wear appropriate PPE (i.e. long pants, eye protection, dust mask); use hearing protection; keep loose clothing, jewelry, hair, and gloves out of grinder and away from the blades. To increase the longevity of the grinder when grinding large volumes, it is recommended to grind samples to 6 mm before grinding to with a cyclone mill to 1 mm.

Order of operations:

1) Remove the original glass jar, if in place, and make sure that the threads on the machine are clear of any debris before attaching the thread portion of the adaptor to the discharge outlet.
2) Twist adaptor onto machine in place of the jar (Fig. 3).
3) Place a sample bag over the adaptor and attach tightly to adaptor at either groove. Depending on bag size/type, either by friction, wire closure or an elastic band can be used to attach to the adaptor at the grooves (Fig. 3).
4) Turn on the grinder.
5) Check for air leaks along where the adaptor is attached to the machine as well as along the area where the sample bag is attached to the adaptor. Using one’s back of the hand is quick and effective way to test for air leaks. This leak check should be performed each time a new bag is attached, and a new sample is ground.
6) Grind sample according to the machine operating manual.
7) Remove the sample bag from the cone. The cone adaptor *does not* need to be removed.
8) To cut down on sample cross contamination on smaller samples, thorough pressurized air cleaning should be done to the entire grinder. Do not use pressurized air on yourself or others. The adaptor *does not* need to be removed between samples, just make sure that pressurized air flows through the machine and out the adaptor.
9) Repeat steps 5–8 for each new sample.

## 7 Validation and characterization

The grinder adaptor cone was designed to increase sample preparation efficiency. A small test was conducted to validate time and sample retention. The standard protocol for the laboratory calls for samples to be ground to 6 mm using a blade type mill, then ground to 1 mm using the Foss CT 293 Cyclotec impeller abrasive grinder. Grinding samples to 6 mm first, allows larger samples to be appropriately homogenized and subsampled as well as reduces wear and tear on the CT 293. Therefore, 60 small (6 g) 6 mm alfalfa samples were weighed out. Thirty samples were ground using the jar attachment as per factory instructions and 30 samples were ground using the adaptor. All samples were transferred into the final storage container, then weighed again to determine sample loss. The process was timed from the initial start of grinding until the closure of the storage container and cleaning of the machine for the next sample was completed. An additional time point was measured at when grinding was complete to determine if grind time was impacted by the adaptor. The validation test used two different individuals rotated between grinding of each sample to introduce the type of variation usually found when a laboratory employs multiple individuals that rotate various tasks and reduce a change of speed that may occur when an individual performs a repetitive task.

The use of the adaptor reduced the amount of sample loss by 5 % (p > 0.0001) (Table 1). While the sample loss using the jar was minimal, as sample volumes get smaller the reduction of loss can become profound. The use of the adaptor reduced both grinding and handling time (p > 0.0001) for a total time savings of 54 s per sample which is a 27 % reduction in time for an individual sample preparation. The reduction in grinding time was unexpected but might be related to the shape of the adaptor increasing air flow.

**Table 1:** Comparison of means for sample preparation between the jar and adaptor. N = 60.

	Jar		Adaptor		Savings
	*Mean*	*SD*	*Mean*	*SD*	
Total time (sec)	185	16	131	15	−54 ***
Grinding (sec)	79	19	58	11	−21 *
Handling (sec)	106	14	73	7	–33 ***
Loss per sample (%)	16	3	11	2	−5 ***

Significance of p-value: *** = 0.0001, ** = 0.001, * = 0.01.

The laboratory has used the device on over 4000 small plant samples (< 15 g) since the design was perfected, which has translated into a time savings of over 430 min. No wear on the part has been seen nor has the adaptor had to be tightened due to vibrations.

In conclusion, this device, while simple, increases the efficiency of sample grinding and transfer of 1 mm material by 27 %. This adaptor design could be adapted to fit any brand of grinder by altering the attachment point.

Ethics statements.

This work was supported by the 10.13039/100007917U.S. Department of Agriculture, Agricultural Research Service. Mention of any trade names or commercial products in this article is solely for the purpose of providing specific information and does not imply recommendation or endorsement by the U. S. Department of Agriculture. 10.13039/100000199USDA is an equal opportunity provider and employer, and all agency services are available without discrimination.

## CRediT authorship contribution statement

**Otto Heuschele:** Writing – review & editing, Writing – original draft, Methodology, Conceptualization. **R. Ted Jeo:** Writing – review & editing, Validation, Investigation, Data curation, Conceptualization. **D. Jo Heuschele:** Writing – review & editing, Writing – original draft, Supervision, Project administration, Funding acquisition.

## Declaration of competing interest

The authors declare that they have no known competing financial interests or personal relationships that could have appeared to influence the work reported in this paper.
